# Understanding the Structural Evolution of Single Conjugated Polymer Chain Conformers

**DOI:** 10.3390/polym8110388

**Published:** 2016-11-03

**Authors:** Adam J. Wise, John K. Grey

**Affiliations:** Department of Chemistry and Chemical Biology, University of New Mexico, Albuquerque, NM 87131, USA; wise.adam.jay@gmail.com

**Keywords:** single molecule spectroscopy, conjugated polymers, emissive conformers

## Abstract

Single molecule photoluminescence (PL) spectroscopy of conjugated polymers has shed new light on the complex structure–function relationships of these materials. Although extensive work has been carried out using polarization and excitation intensity modulated experiments to elucidate conformation-dependent photophysics, surprisingly little attention has been given to information contained in the PL spectral line shapes. We investigate single molecule PL spectra of the prototypical conjugated polymer poly[2-methoxy-5-(2-ethylhexyloxy)-1,4-phenylenevinylene] (MEH-PPV) which exists in at least two emissive conformers and can only be observed at dilute levels. Using a model based on the well-known “Missing Mode Effect” (MIME), we show that vibronic progression intervals for MEH-PPV conformers can be explained by relative contributions from particular skeletal vibrational modes. Here, observed progression intervals do not match any ground state Raman active vibrational frequency and instead represent a coalescence of multiple modes in the frequency domain. For example, the higher energy emitting “blue” MEH-PPV form exhibits PL maxima at ~18,200 cm^−1^ with characteristic MIME progression intervals of ~1200–1350 cm^−1^, whereas the lower energy emitting “red” form peaks at ~17,100 cm^−1^ with intervals in the range of ~1350–1450 cm^−1^. The main differences in blue and red MEH-PPV chromophores lie in the intra-chain order, or, planarity of monomers within a chromophore segment. We demonstrate that the Raman-active out-of-plane C–H wag of the MEH-PPV vinylene group (~966 cm^−1^) has the greatest influence in determining the observed vibronic progression MIME interval. Namely, larger displacements (intensities)—indicating lower intra-chain order—lower the effective MIME interval. This simple model provides useful insights into the conformational characteristics of the heterogeneous chromophore landscape without requiring costly and time-consuming low temperature or single molecule Raman capabilities.

## 1. Introduction

The existence of multiple conformers in conjugated polymers has important implications for material performance and can be revealed most straightforwardly from optical absorption and photoluminescence (PL) spectra [1,2,3,4,5,6,7,8]. Understanding the structural (conformational) attributes of chromophores in their ground and excited electronic states is necessary for obtaining molecular-level structure–function information. One of the best studied systems displaying multiple conformers is the prototype conjugated polymer poly[2-methoxy-5-(2-ethylhexyloxy)-1,4-phenylenevinylene] (MEH-PPV). Seminal studies at the single molecule level exposed bimodal PL energy distributions due to incomplete energy funneling within single chains [3,6,9,10,11,12]. So-called “blue” and “red” emitting forms of MEH-PPV can be isolated where the former arises from solution-like conformations (i.e., defect coil) and the latter more closely resembling collapsed chains in bulk films (i.e., defect cylinder) [13]. Interestingly, the energetic separation between blue and red forms is almost exactly one vibronic PL interval which may arise from vibronic or excitonic coupling mechanisms. Despite the extensive studies of the bimodality and apparent accidental vibronic interval energy separation phenomena in MEH-PPV and many other polymer systems, investigations into the structural attributes of these emitters and the extent of geometric rearrangement between ground and emitting states has received much less attention.

In their detailed study of transformations between emitting species, Köhler et al. reported a second order phase transition between disordered (blue) and ordered (red) forms at ca. 80 K in MEH-PPV chains dispersed in a glassy matrix [14]. This transition is proposed to become favorable due to cooperative electronic stabilization from increased planarity of chain segments which then leads to dominant intra-chain exciton coupling and J-aggregate type excitons. Unlike crystalline π-stacked polymer aggregates, such as in the well-studied poly(3-hexylthiophene) (P3HT) system, inter-chain interactions should be minimal in MEH-PPV due to large steric hindrance effects of the branched alkoxy side group. The intra-chain view of the red form excitons was also confirmed by Basché and coworkers who showed that inter-chain aggregation is limited in these chromophores [15]. These findings support the assignment of a J-aggregate type exciton coupling in the red form indicating a tendency of MEH-PPV chains to adopt highly ordered rod-like structures [16]. However, the stochastic nature of polymer solution processing and polydispersity effects makes it difficult to reliably control conformational aspects of polymer chains in thin films implying that the likelihood of forming ordered J-aggregate type chain conformations is relatively small. On the other hand, ensemble emission spectra line shapes from bulk films tend to be dominated by the red form because of efficient excitation energy transfer from more plentiful, higher energy blue sites [17]. The exciton coupling characteristics can be tuned further in the condensed phase by applying post-processing thermal annealing treatments causing an apparent change in lineshape from J- to H-type aggregation where the 0-0 transition intensity is diminished relative to the 0-1 sideband [18,19]. When films are annealed for longer times above the glass-transition temperature, PL line shapes more closely resemble excimers [20] which can subsequently be converted back to J-type emission by exposing films to solvent vapors (see below).

In order to gain molecular perspectives of the evolution of structure–function relationships, Barbara and coworkers introduced a single molecule spectroscopy approach combined with in-situ solvent vapor annealing (SVA) treatments [16,21,22]. These authors used excitation polarization modulation techniques to study the relative order of single chain segments by rotating the excitation polarization angle from 0° to 180° and recording modulation depths (*M*) of single chain PL intensity transients. As shown previously by Hu et al. typical *M* values of single MEH-PPV chains cast from solutions vary from ~0.1–0.8 with the average around 0.6 [23]. Larger *M* values indicate higher order within the chain (i.e., aligned segments) and more closely resemble single dipole emitters whereas smaller *M* values arise in multi-chromophoric emitters with little order between segments and, consequently, no preference to excitation polarization direction [24]. Traub et al. extended this work by incorporating strategically placed defects, namely, terphenyl groups linked via ortho and para with monomers, to direct chain folding and therefore make correlations between *M* values and the emitting form [16]. They showed para-substituted MEH-PPV chains have large *M* values (~0.8) in contrast to ortho-substituted chains that had much smaller *M* values (~0.2) following SVA treatments. Moreover, PL spectra of the former tend to exhibit emission from the red form while the latter are almost exclusively blue-type emitters.

In addition to PL maxima shifting in energy, the line shapes of solution and solid forms of PPV derivatives change significantly depending on their specific conformations (processing conditions) [19]. In particular, the vibronic interval can vary between ~1220 and 1280 cm^−1^ for solutions compared to ~1340–1400 cm^−1^ in solid forms (i.e., films and nanoparticles) [19]. These intervals do not match any ground state vibrational frequency and instead arise from the coalescence of several displaced vibrational coordinates. Thermal broadening effects mask individual transitions and only one effective interval is observed, which is known as the missing mode effect (MIME) [19,25,26]. Because the individual vibrational displacements (Δ) also depend on the chain conformation and polarizability of the surrounding medium, the MIME interval can be a sensitive reporter of polymer conformation and planarity of chain segments. In a related study, we found one PPV-type polymer backbone mode was particularly sensitive to the structural characteristics, namely, the out-of-plane C–H wag mode of the backbone vinylene group (~961–966 cm^−1^) [19]. Pre-resonance Raman spectra of solutions and thin films were used to estimate vibrational mode-specific displacements which can then be used to fit the broadened PL lineshapes [25]. Because the C–H wag is forbidden in a planar backbone geometry, increased intensities (activity) indicate larger emitting state displacements and a more distorted backbone structure. Interestingly, similar effects were observed upon addition of large amounts of a fullerene derivative, which exhibit preferential interactions with the vinylene group due to intercalation [27].

Here, we study MEH-PPV single molecules in various host matrices and with SVA treatments to tune conformational characteristics of the polymer backbone. We show that the vibronic progression intervals vary depending on the emitting form and can be explained by the MIME model. By varying the molecular weight of the host matrix (i.e., viscosity and solvent evaporation time) it is possible to preferentially select either blue or red forms that are correlated to the conformational order characteristics. For example, dispersing MEH-PPV into an ultra-high MW polystyrene matrix (~2 MDa) at high concentration induces folding of the chain and spectra almost entirely resemble the red form with a relatively narrow peak energy distribution. Either lowering the matrix concentration or switching to a lower MW results in the “normal” bimodal behavior. We show that subsequent SVA treatments in these lower MW hosts lead to collapse into the red form, similar to high MW hosts. Additionally, we report the emergence of a minority lower energy emitter separate from the typical blue and red species in low MW polystyrene hosts. These “ultra-red” species overlap energetically with excimer-like transitions in thermally annealed bulk films although they possess resolved vibronic structure with PL line shapes resembling blue forms. Adapting the simple MIME line shape analysis to MEH-PPV single molecules in different environments sheds new light on the structural qualities of emitter chromophores beyond chain conformational characteristics determined from polarization studies.

## 2. Experimental

Dispersions of MEH-PPV (Polymer Source; ~950 kDa, PDI = 2.2) single molecules were prepared by serial dilution of 1 mg/mL chloroform solutions into polystyrene hosts of varying molecular weight and concentration. The MEH-PPV stock solution was stirred overnight with a stir-bar inside a nitrogen glovebox and then dispersed into chloroform solutions of 50 kDa and 2 MDa polystyrene GPC standards (Aldrich, St. Louis, MO, USA). Polystyrene solution concentrations were 2 and 20 mg/mL and spin cast onto rigorously cleaned glass coverslips inside the glovebox at 1000 rpm for two minutes. An aluminum overcoating was applied by thermal evaporation following spin-casting and samples were stored under nitrogen until approximately one hour before use. Solvent vapor annealing was performed on some samples prior to aluminum coating by placing the films in a sealed chamber at room temperature with a reservoir of toluene.

Single molecule imaging and spectroscopy were carried out using a confocal laser microscope described in detail previously [19]. Dilute thin film samples were illuminated with the 488 nm line of an Argon/Krypton laser (Melles Griot, Albuquerque, NM, USA), and excitation power densities used were approximately 100 W/cm^2^. Scattered excitation light was removed by a long-pass edge filter (Semrock, Rochester, NY, USA) and emitted was imaged onto an avalanche photodiode (Perkin Elmer, Waltham, MA, USA) for imaging or the slits of a CCD spectrograph (Andor, Belfast, UK) for measuring spectra. All measurements were performed under an inert atmosphere by placing the sample in a sealed nitrogen flow cell. A representative single molecule PL image is shown in Supplementary Materials (Figure S1) and typical areal densities were ~0.2 molecule/μm^2^.

Time correlated single photon counting (TCSPC) measurements were carried out on the same microscope system using a fast response avalanche photodiode and photon counting computer board (Becker-Hickl, Berlin, Germany). Excitation was provided by a 450 nm pulsed diode laser (Edinburgh Instruments, Edinburgh, UK) and at a repetition rate of 20 MHz. The instrument response function was recorded using scattered excitation light from a cleaned glass coverslip. Latex dye beads (Duke Scientific, Duke, NC, USA) were used to calibrate the spatial resolution and response of the instrument.

## 3. Results and Discussion

Effect of Host Matrix Molecular Weight on Chain Conformation.

**Figure 1:**
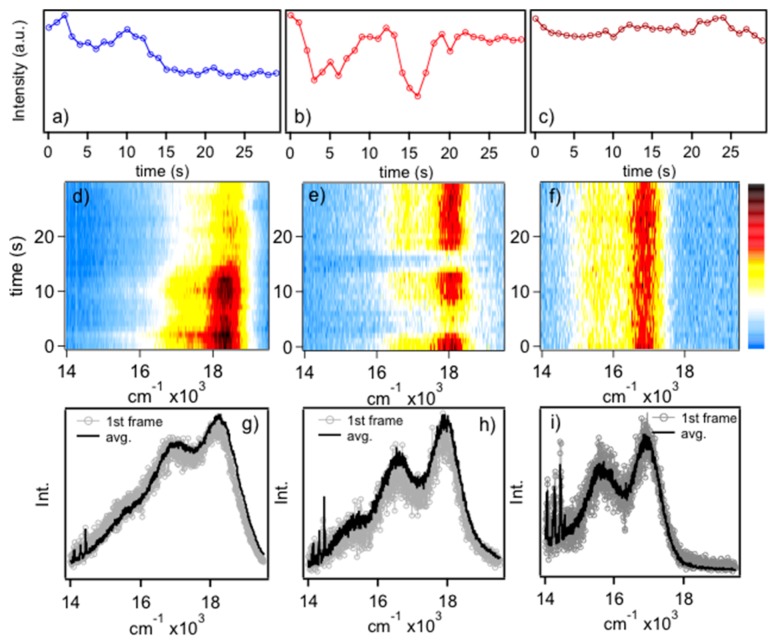
Examples of: PL (photoluminescence) integrated intensity transients (**a**–**c**); PL spectra transients (**d**–**f**); and PL spectra (**g**–**i**) of the first frame (gray) and average (black) of the “blue”, “red” and “ultra-red” emitting forms of MEH-PPV (poly[2-methoxy-5-(2-ethylhexyloxy)-1,4-phenylenevinylene]) single molecules. Spike features appearing on the lower energy tail of the PL line shape correspond to plasma lines from the laser excitation source.

MEH-PPV single molecule emitting forms are first categorized then PL line shapes are next analyzed to determine structural characteristics (i.e., vibrational displacements) of these chromophores. Figure 1 shows representative PL transients (Figure 1a–c), spectral dynamics (diffusion) (Figure 1d–f) and comparisons of averaged and first frame spectra (Figure 1g–i) of MEH-PPV chains dispersed in polystyrene hosts. Spectra can be classified according to their energies, which correspond to blue, red and “ultra-red” emitters and the full distributions are presented and discussed in the following. Assignment of emissive conformers was based on the PL energy of the electronic origin (*E*_0-0_) and sub-ensemble PL spectra were generated by averaging spectra within their respective distributions (vide infra). In general, blue emitters typically show step-wise flickering whereas red emitters show discrete on/off flickering over the timescale of the measurements. These behaviors are indicative of multi-chromophoric and single chromophore emission, respectively, and reflect the conformational characteristics of the individual polymer chains [11]. Namely, blue emitters originate from solution-like conformations (i.e., defect cylinder) where energy transfer between segments is relatively inefficient probably due to poor dipole–dipole orientation factors. Conversely, red emitting chains exist in collapsed conformations that favor rapid energy migration to as little as a single emitting site and reversible photo-oxidation results in the observed telegraphic (on–off) flickering behavior. As mentioned earlier, these chromophores possess J-aggregate exciton characteristics, which require elongated and planarized segments [28,29]. Ultra-red emitters show high photo-stability with no distinct flickering behavior. Very little spectral diffusion is observed for the single molecule spectra in this study, which is expected for the smaller excitation power densities used. It is possible, however, that spectral fluctuations could occur on time scales faster than the PL spectra acquisition times used. However, no significant deviations of energy maxima are observed over the course of the measurement consistent with minor contributions from spectral diffusion. We now consider the structural and conformational characteristics of these emitters and the how they evolve between each form.

**Figure 2:**
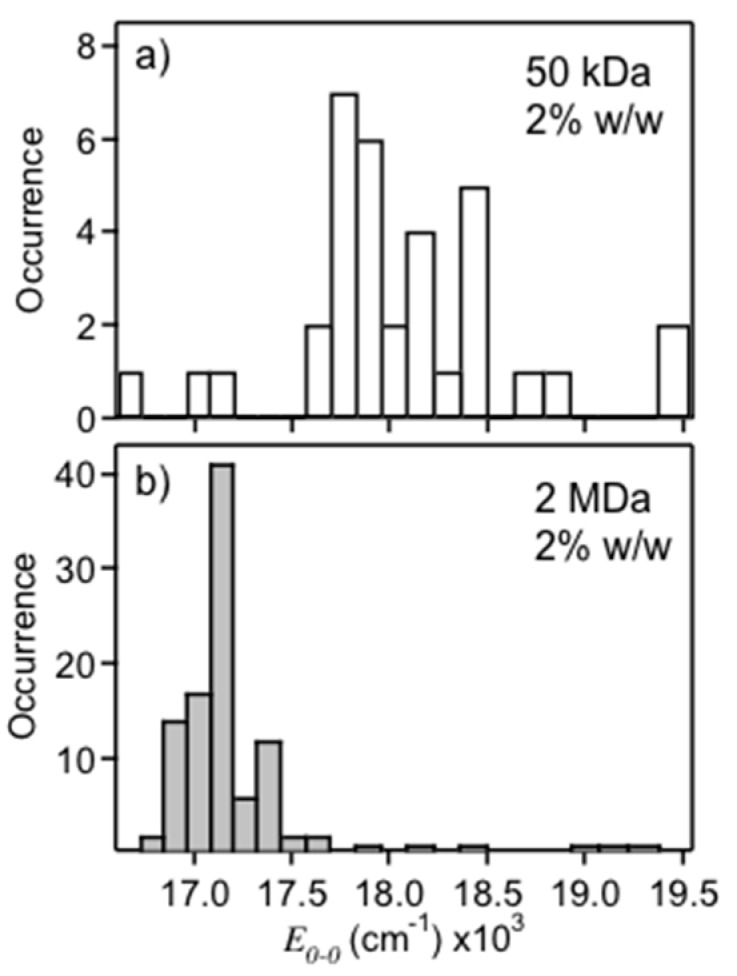
Histograms of the PL *E*_0-0_ maxima for MEH-PPV single molecules dispersed in: 50 kDa (**a**); and 2 MDa (**b**) polystyrene.

There has been ongoing debate over the structural characteristics of the red emitting form of MEH-PPV, which mostly correspond to emissive traps in bulk films (see Supplementary Materials, Figure S2). Although single molecule spectroscopy studies have shown that this species does not require more than one chain to exist, much of the detailed information in PL line shapes remains to be harvested to construct more meaningful pictures of their structural qualities beyond inferences and expectations from exciton coupling theory. In particular, assessing the extent of geometrical displacements between ground and excited electronic states as well as the vibrations involved is especially important for understanding and controlling charge and energy transfer processes. To this end, we evaluate MEH-PPV single molecules dispersed in polystyrene hosts of varying molecular weight and apply post processing SVA treatments to selectively tune chain conformations. Approximately 50–100 spectra and transients are recorded for each sample and compared by plotting histograms of the energy of the electronic origin transition (*E*_0-0_). This parameter is more meaningful than the peak energy maximum (*E*_max_) to compare chain conformational characteristics since mixed emitters (i.e., blue and red emitters within the same molecule) tend to bias the distributions typically towards the red emitters. For this reason, we exclude mixed emitters from the analysis, which only comprise about 15% of the total molecules studied. Spectra from each host were recorded under the same conditions and we found no correlation between brightness (integrated PL counts) and type of emitter. This aspect is essential for comparing single molecule statistics since aggregates can often overwhelm single chain emission. Figure 2 shows PL *E*_0-0_ histograms for MEH-PPV single molecules dispersed in 50 kDa (Figure 2a) and 2 MDa (Figure 2b) polystyrene hosts each at a concentration of 2% (*w*/*w*) in chloroform. *E*_0-0_ distributions of the 50 kDa samples show primarily emission from the blue form (~18,200 cm^−1^), whereas the 2 MDa samples show emission exclusively from the red form (~17,100 cm^−1^). The spread in PL energies is also narrower for the latter (~100 cm^−1^ vs. ~200 cm^−1^) indicating less disorder. The ability to select either emitting form likely originates from host–molecule interactions and solvent evaporation times. The number averaged MW of MEH-PPV chains used in this study was ~950 kDa which appears to be better solvated in the lower MW host thus favoring mostly solution-like conformations (blue emitters). Chang et al. also found that high MW MEH-PPV chains are well solvated in small molecule liquid crystals, consistent with our observations [30]. It is also important to note the viscosity of the 2 MDa polystyrene sample was considerably higher than the 50 kDa sample leading to longer solvent evaporation rates. MEH-PPV chains likely have a longer time to adopt their thermodynamically favorable structure (i.e., collapsed conformations) in these hosts thus mimicking SVA treatments (see below) due to swelling of the matrix.

**Figure 3:**
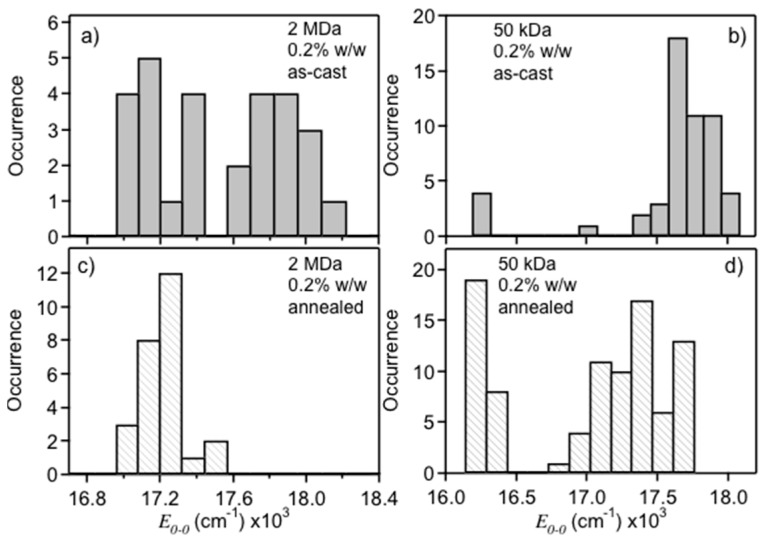
Comparison of PL *E*_0-0_ maxima for MEH-PPV single molecules in dilute: 2 MDa (**a**,**c**); and 50 kDa (**b**,**d**) polystyrene dispersions before and after solvent vapor annealing, respectively.

To test this hypothesis, both polystyrene hosts were cast at lower concentrations (0.2% *w*/*w*) to produce thinner films (~40 nm). Figure 3a,b shows *E*_0-0_ histograms for the thin 50 kDa and 2 MDa dispersions. The thinner 50 kDa samples behaved similarly to the thicker films generated, whereas the 2 MDa samples showed considerably more blue emitters in thinner films giving rise to the familiar bimodal emitter distribution [3,9]. To determine if the blue emitters could be converted to the red from after casting, samples were subjected to SVA treatments inside a glovebox then sealed with aluminum under a base pressure of ~10^−7^ mbar. Figure 3c,d displays *E*_0-0_ histograms for SVA treated thin films of 2 MDa and 50 kDa samples, respectively. The bimodal distribution collapses to only the red form in both films, similar to the thicker samples of the 2 MDa matrix, indicating that indeed the higher MW hosts (viscosities) have similar outcomes as SVA treatments. Thin annealed samples of the 50 kDa polystyrene host show an overall red-shift due to an increased number of red species, but an additional species emerges approximately 1100 cm^−1^ further to the red. A small amount of this form was observed in as-cast films (<10%) that increases up to ~35% upon SVA treatment. These ultra-red emitters appear almost exclusively in the 50 kDa host and may result from surface perturbations with a greater fraction of molecules in contact with the substrate. Previous work from Barnes and coworkers also report a similar species when MEH-PPV molecules are cast directly on glass suggesting a surface-induced folding of the polymer chain [31,32]. The similarity of this emitter with bulk films may further indicate these species are aggregates although average intensities were not significantly brighter. We next analyze PL line shapes of each emitting form to generate deeper insights about their structural and conformational attributes and how these affect exciton coupling and energy transfer.

### 3.1. Extending the MIME Description to Single MEH-PPV Molecules

**Figure 4:**
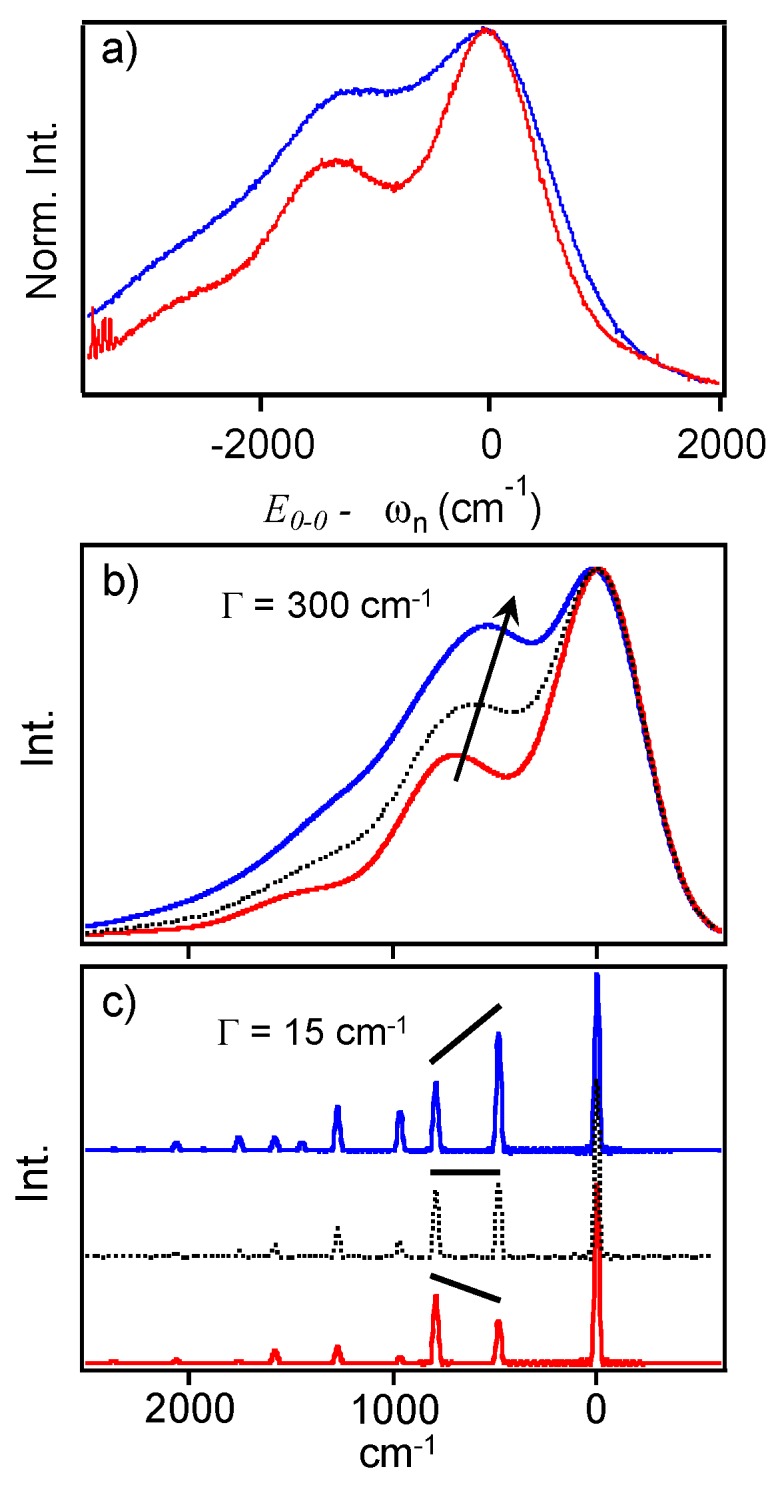
(**a**) Sub-ensemble single MEH-PPV chain PL spectra from “red” and “blue” forms (red and blue traces, respectively) for both experimental and simulated PL spectra; (**b**) Calculated PL spectra of MEH-PPV sub-ensemble emitters using the simplified two-mode model with a damping constant of 300 cm^−1^. Arrow indicates shift of the apparent vibronic interval; (**c**) Calculated PL spectra of MEH-PPV emitters using the simplified two-mode model with a damping constant of 15 cm^−1^. Black lines are included as a guide for the eye and illustrate underlying trends of the intensity distributions for the broadened intervals in (**b**).

Although the bimodal emission phenomenon is well-known for single MEH-PPV molecules, surprisingly little attention has been paid to the line shapes which contain useful information surrounding the structural characteristics of chromophores. Figure 4a shows sub-ensemble averaged spectra for blue and red emitters generated from thin 2 MDa as-cast films. Similar sub-ensemble averaged spectra are included in the Supplementary Materials (Figure S3), which were generated from 50 kDa films. To qualitatively illustrate the difference in PL lineshapes for each form, spectra are overlapped at their *E*_0-0_ values. Comparison of these averaged line shapes show noticeable differences, namely, smaller Huang–Rhys factors and vibronic frequency intervals in the red form. These features are consistent with observations of MEH-PPV and related derivatives in solution and solid forms, where the latter always have a larger progression interval. Furthermore, these differences do not arise from significant variations in the refractive index, which is nearly constant in this energy range. The vibronic spectra presented herein display clear manifestations of the MIME where several displaced vibrational modes coalesce into one effective interval. This MIME is more easily observed in individual single molecule spectra since ensemble averaging for each form tends to broaden lineshapes making it less distinct. Analysis of each spectrum can reveal the intrinsic heterogeneity within each emitting form that is otherwise obscured in the ensemble data.

We previously used up to five modes to fit PL spectra of a related derivative, MDMO-PPV, in various forms. It was demonstrated that individual displacements vary substantially depending on the specific sample form, which, in turn, dictate the effective progression interval [19]. Vibrational displacements were estimated from pre-resonance Raman intensities using the Savin formula, *I*_1_/*I*_2_ = (ω_1_Δ_1_)^2^/(ω_2_Δ_2_)^2^. Importantly, the largest change in displacement between solution and solid forms occurred in the out-of-plane C–H wag mode of the PPV vinylene linkage (~961–966 cm^−1^) [19]. While the relative Raman intensities of this mode are typically small, its displacement, according to the Savin formula, can be comparable or larger than more intense, higher frequency modes (i.e., symmetric CC stretches of the phenyl group, ~1580 cm^−1^). To illustrate the effect of varying the displacement of the C–H vinyl wag on the MIME interval, we use a simple two-mode model, namely, the ~1580 and 966 cm^−1^ modes, which comprise the upper and lower frequency range from our previous MIME analysis of MDMO-PPV [19].

PL lineshapes are calculated using the time-dependent theory of spectroscopy of Heller and coworkers [33]. In this approach, a Gaussian wavepacket (φ) is projected vertically from the lowest vibrational level of the initial state (emitting state) to the final state (ground state). The wavepacket evolves in time according to the time-dependent Schrödinger equation. The emission intensity is then described by:

(1) $$I\left(\omega \right)\propto \omega^{3}\int_{-\infty }^{\infty }\langle \varphi |\varphi \left(t\right)\rangle \exp\left(i\omega t\right)dt$$

where ω is the frequency of the emitted photon and the multi-mode, time-dependent wavepacket overlap autocorrelation function, 〈φ|φ(t)〉, is given by:

(2) 〈φ|φ(t)〉=exp{−∑k[12Δk2(1−exp(−iωt))− iωkt2]− iE0−0tℏ− Γ2t2}

where Δ*_k_* is the dimensionless vibrational displacement for the *k*-th mode, *E*_0-0_ is the energy of the electronic origin transition (cm^−1^), and Γ is a phenomenological damping factor (cm^−1^) that determines observed vibronic linewidths. This expression can be solved analytically provided that the ground and emitting state potential surfaces are well described by undistorted harmonic oscillators, i.e., no change in frequency between ground and emitting states, and the transition moment is constant.

Figure 4b shows calculated PL spectra from the two-mode model using Equations (1) and (2) illustrating the effect of the low frequency C–H wag displacement on the MIME interval. Initially, displacements of both modes are set to 1.0 and spectra are calculated for large (Γ = 300 cm^−1^) and small (Γ = 15 cm^−1^) linewidths (black dotted traces). Decreasing the C–H wag mode displacement to 0.75 results in lower intensities and a smaller contribution to the observed interval in the broadened spectrum. In this case, the MIME increases to ~1400 cm^−1^ (red solid traces) from ~1300 cm^−1^. On the other hand, increasing the displacement to 1.25 shifts intensity away from the 1580 cm^−1^ mode causing a smaller effective progression interval of ~1150 cm^−1^ (blue solid traces). Although actual vibronic lineshapes contain more displaced modes, spectra from this simple model effectively demonstrate the impact of Franck-Condon active low frequency vibrations and, more importantly, the sensitivity of the effective vibronic interval on their displacements. It is also apparent that the frequency, displacement and damping factor govern the observed MIME interval, which can never be larger than the highest frequency mode [25]. As the temperature decreases, individual vibronic transitions become resolved and the MIME disappears, which can be replicated here by lowering the damping factor, Γ. We show that tracking changes in the MIME interval from room temperature single molecule spectra is an effective means for understanding conformational attributes of single MEH-PPV chains without the need for more time-consuming and expensive low temperature measurements. Example low temperature (ca. 20 K) PL spectra of red and blue MEH-PPV emitters are shown in the Supplementary Materials using a similar model albeit with an additional low frequency mode to fill in regions between the main progression interval. Here, vibronic maxima are still mostly “smeared” together but these spectra are useful for illustrating improved vibronic resolution.

**Figure 5:**
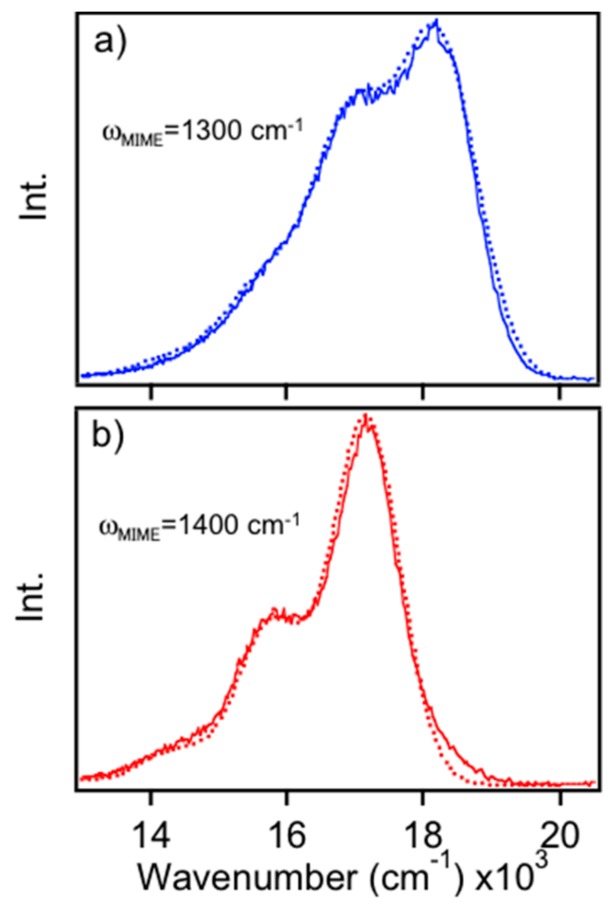
Representative single chain MEH-PPV PL spectra for: blue (**a**); and (**b**) red emitting forms. MIME progression intervals are included.

**Figure 6:**
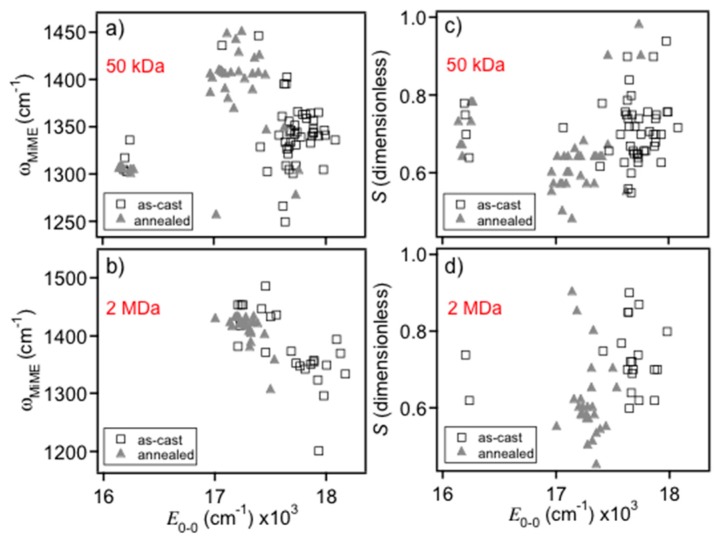
Histograms of the MIME progression interval and Huang–Rhys factor (S) for MEH-PPV chains dispersed in: 50 kDa (**a**,**c**); and 2 MDa (**b**,**d**) polystyrene before and after solvent vapor annealing.

Unlike previous MIME studies on concentrated samples, it is not possible to measure single molecule Raman spectra for estimating individual vibrational displacements. Instead, we use a simple effective coordinate model to fit the single molecule MIME spectra that replicates the MIME interval. Fit spectra are generated for each single molecule PL spectrum and the parameters are compared between each emitting form. Figure 5a,b shows representative fits of blue and red forms from 0.2% *w*/*w* 2 MDa polystyrene as-cast and annealed samples, respectively (similar sub-ensemble fits for the 50 kDa samples are included in the Supplementary Materials, Figures S3 and S4). Despite lower signal-to-noise ratios of single molecule spectra, the fitting procedure is able to distinguish differences in observed vibronic intervals between each form. Figure 6 shows scatter plots of the MIME frequency (Figure 6a,b) and apparent Huang–Rhys factor (*S*=12Δk2; c,d) generated from the fits which show distinct bimodality behavior similar to *E*_0-0_ histograms of Figure 2 and Figure 3. The average MIME frequency for blue-emitting MEH-PPV chains was ~1350 ± 60 cm^−1^ compared to ~1430 ± 40 cm^−1^ for red-emitters consistent with our previous work on solution and solid forms of MDMO-PPV. Average apparent *S* values are also smaller for the red form, in agreement with recent findings that the red form behaves as a J-aggregate [15]. It is important to stress that this parameter is used to reproduce the overall line shape profile (i.e., effective vibronic intervals) meaning that both exciton and vibronic coupling are collectively expressed in fitted *S* values. 

Differences in the observed MIME intervals between red and blue forms are proposed to originate chiefly from variations in displaced low frequency modes, most notably the out-of-plane C–H wag at ~966 cm^−1^. Based on our previous work, this mode showed the largest change between solution and solid polymer forms, which is reasonable since larger intensities (displacements) are expected for torsionally disordered chains. As demonstrated in Figure 4b,c, larger displacements in this mode shift intensity away from higher frequency modes resulting in a smaller observed MIME interval range of ~1220–1320 cm^−1^. Displaced low frequency modes effectively “fill in” regions between vibronic peaks and, while a similar effect can be obtained by increasing the linewidth broadening parameter, Γ, the fit spectrum no longer matches with the linewidth of the 0-0 electronic origin transition. On the other hand, the larger observed MIME interval in the red emitting form implies a smaller C–H mode displacement where intensity is shifted toward the higher frequency modes (i.e., the ~1580 cm^−1^ mode), as seen in Figure 4b,c. This simple analysis of single chain PL lineshapes reveals useful insights into the conformational characteristics of each emitting form far beyond simply categorizing emission energies. Namely, the lower progression interval observed in the blue form indicates larger displacements of the out-of-plane C–H wag and, hence, larger torsional distortions of the backbone conformation. This feature is also consistent with the apparent shorter conjugation lengths of the chromophore segments (i.e., torsional induced exciton localization). Dispersing single molecules in a heavy matrix or applying SVA treatments, results in planarization of chain segments due to swelling of the matrix thus allowing chains to self-fold into more ordered, rod-like or collapsed structures [21,22,23,24]. The increase of chain planarity lowers the C–H wag displacement resulting in a larger MIME interval as seen in PL line shapes of the red MEH-PPV form. Overall, interpreting spectral changes in terms of the MIME model yields useful insights into the conformational characteristics of the polymer backbone segments as well as the structural factors that change upon conversion between these different emitting forms.

It is also necessary to consider the possibility that the difference in MEH-PPV single chain line shapes between different emitting forms arises from multiple electronic origins (emitting chromophores). For example, Basché and coworkers reported single chain MEH-PPV spectra dispersed in different host matrices displaying multiple origins at low temperatures (ca. 1.2 K) [34]. These transitions are typically spaced close enough so that linewidth broadening at higher temperatures should cause coalescence into an effective progression interval. We found comparatively few spectra displaying evidence of multiple emitters that were excluded from the analysis mainly because these line shapes tend to show spectral diffusion due to photo-oxidation of individual chromophores of different energies and energy funnels [9,35]. Since spectral diffusion was small and virtually no spectra showed switching between blue or red emitters, we conclude that multiple origins are not responsible for differences in MIME intervals. We also used lower excitation intensities (i.e., 100 W/cm^2^ vs. 2 kW/cm^2^) thus lowering photo-oxidation yields and providing better photo-stability and small spectral diffusion. This is especially important for tracking the MIME phenomenon in single molecules because “off” periods limit overall signal-to-noise ratios making it difficult to reliably assess the MIME phenomenon.

### 3.2. Ultra-Red MEH-PPV Emitters

We now return to the appearance of ultra-red emitters for MEH-PPV chains dispersed in the lower MW polytstyrene matrix. These minority emitters become most apparent after annealing and the MIME progression interval in this species is closer to the blue form (~1330 cm^−1^). The latter feature indicates a larger contribution from lower frequency vibrations (i.e., out-of-plane C–H wag) and lower intrachain chromophore order. However, *E*_0-0_ values and good photostability (i.e., absence of flickering in transients) of the ultra-red form are similar to PL spectra of MEH-PPV aggregates. For example, MEH-PPV nanoparticles can be fabricated by rapidly injecting well-dissolved chains into a non-solvent, such as water. PL spectra of these nanoparticle aggregates and the ultra-red form are included in the Supplementary Materials that show very similar line shape characteristics. It has also been demonstrated that these larger particles exhibit no flickering behavior and are typically photostable for long times [36,37]. We conclude that ultra-red emitters are most likely small aggregates of MEH-PPV chains that associated upon SVA treatment.

**Figure 7:**
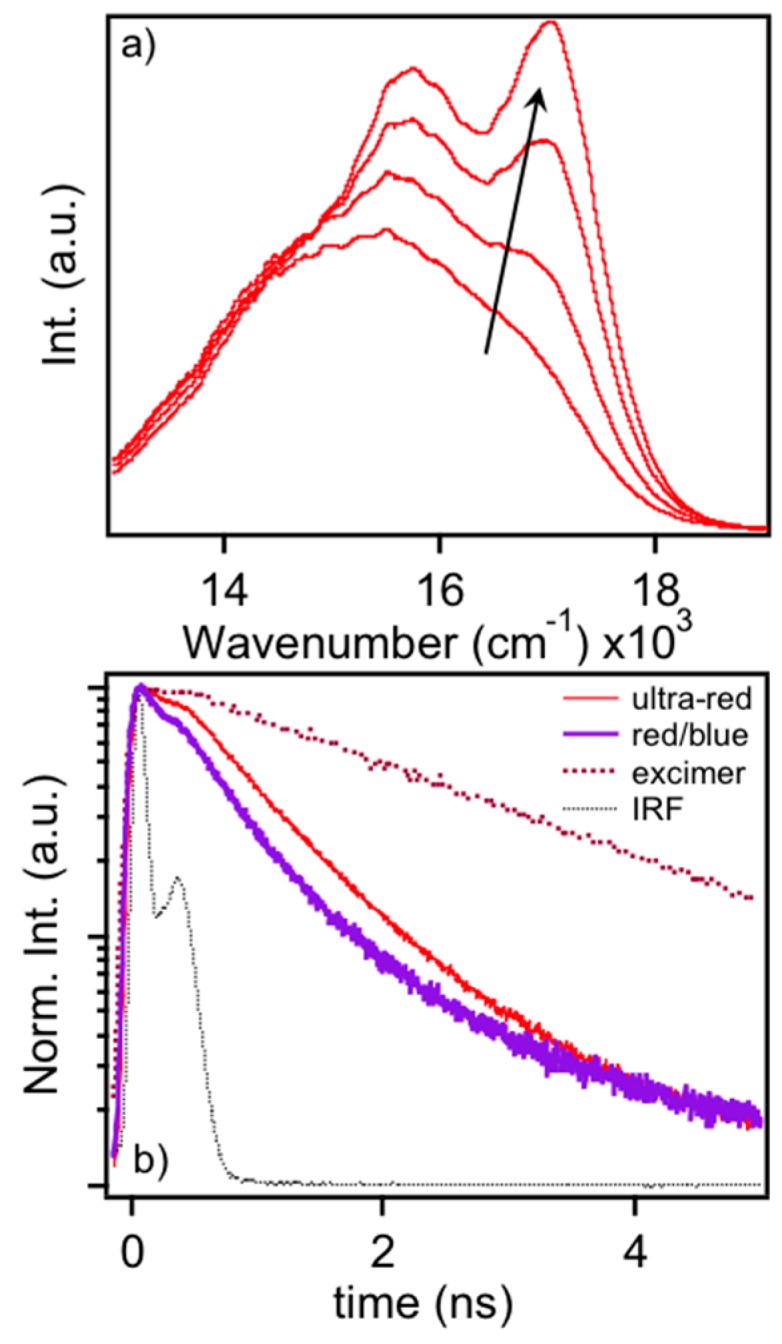
(**a**) PL spectra of an MEH-PPV thin film upon application of solvent vapor annealing; and (**b**) PL decays of single molecule MEH-PPV emitting species. Corresponding PL decay of an annealed thin film displaying excimer type emission is also included.

The similarity of ultra-red MEH-PPV emitters to aggregated or agglomerated MEH-PPV nanoparticles illustrates the rapid convergence of single molecule emitters to the bulk-like PL emission from thin films. Here, rapid energy funneling to minority emissive traps leads to their red-shifted PL line shapes relative to dilute solution phase PL line shape characteristics. Interestingly, application of thermal annealing to MEH-PPV thin films reveals further collapse of this line shape to one resembling an excimer type state, as shown in Figure 7a. Subsequent SVA treatment restores PL line shapes to the characteristic form as seen in aggregates or the ultra-red variant. In the excimer form, there are a greater number of vibrational modes distorted since the excitation is intermolecular in nature. Restoration of the typical PL line shape in the condensed phase indicates these excitons possess greater intramolecular character and, consequently, are more sensitive to chromophore structure and chain conformation.

To further sort out the structural differences as well as those of excitons between typical red/blue forms and the bulk-like ultra-red and excimer forms of MEH-PPV, we use time-correlated single photon counting techniques to study the PL decay dynamics in each form. Figure 7b shows ensemble averaged PL decays of single MEH-PPV molecules. Corresponding PL spectra were measured to correlate the specific emitting form to decay characteristics. We found very little differences between the single molecule blue and red PL forms and average these decays together in Figure 7b, which show decay times of ~300 ps similar to previous work [13]. Average decay times for the ultra-red species were found to be ~500 ps whereas the excimer-like transition in thermally annealed bulk films is ~2 ns. In addition to the increase in PL decay time, the high photo-stability of the ultra-red species indicates multi-chromophoric behavior where virtually no flickering or bleaching occurs within the timeframe of the measurement (>3 min). Combined with PL spectral characteristics, these data clearly show stark differences between the intra- and inter-molecular exciton regimes. 

## 4. Conclusions

In this paper, we demonstrated that analysis of MEH-PPV PL spectral intervals can be related to the emission energy of structural polymorphs. The higher energy “blue” form exhibits conformational characteristics similar to those of the polymer molecules in dilute solutions. In this scenario, large monomer torsional disorder exists leading to larger displacements in the out-of-plane vinyl C–H wag of ~966 cm^−1^ and a lower effective MIME progression interval. On the other hand, the lower energy emitting “red” form possesses greater monomer planarity which lowers the contribution of the ~966 cm^−1^ mode. Larger progression intervals result, which are closer to the dominant in-plane CC symmetric stretching vibrations of the backbone. The appearance of the “ultra-red” emitting form suggests that a minority fraction of chains are capable of π-stacking and the longer PL lifetimes in addition to lower MIME PL intervals. We propose these structures are excimer precursors that become more plentiful when MEH-PPV thin films are thermally annealed for long times.

## Supplementary Materials

The following are available online at www.mdpi.com/2073-4360/8/11/388/s1, Single molecule PL images, sub-ensemble sorted MEH-PPV PL spectra, sub-ensemble PL spectra fits, histograms of fit parameters, representative low temperature MEH-PPV single molecule PL spectra with fits, and comparison between MEH-PPV nanoparticle aggregates and ultra-red sub-ensemble spectra.
