# Supplementary material for: Understanding the Structural Evolution of Single Conjugated Polymer Chain Conformers
Source: Polymers (Basel). 2016 Nov 3;8(11):388. doi: 10.3390/polym8110388 (PMC6432208; doi:10.3390/polym8110388)
Supplement: Supplementary file 1 [file polymers-08-00388-s001.pdf]

Supplementary Materials: Understanding the Structural Evolution of Single Conjugated Polymer Chain Conformers

Adam J. Wise and John K. Grey

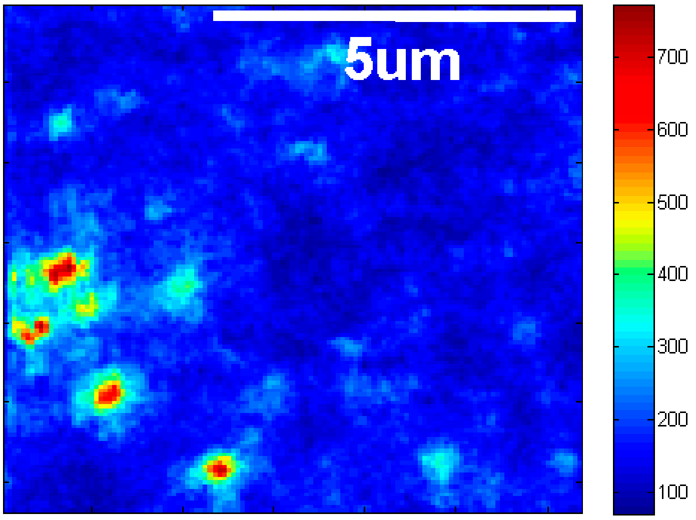

Figure S1. Representative PL image of well dispersed MEH-PPV single molecules in a polystyrene host.

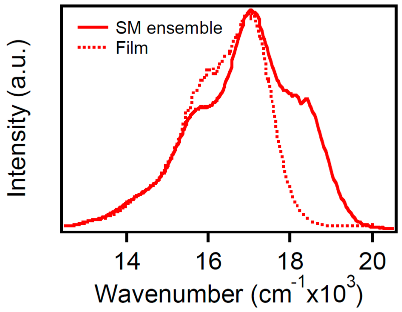

Figure S2. Comparison of ensemble averaged single molecule and bulk film PL spectra of MEH-PPV.

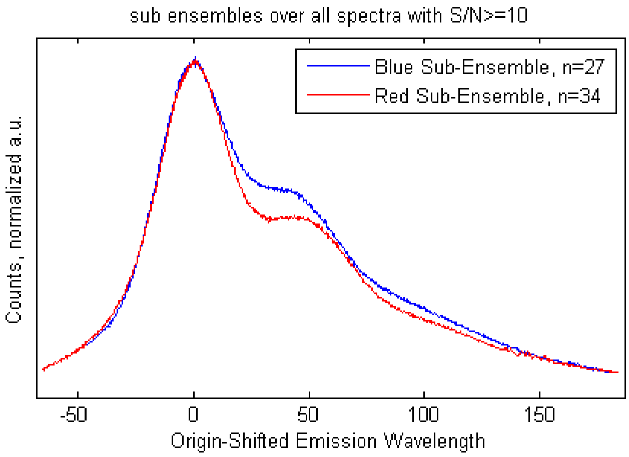

Figure S3. Comparison of blue and red sub-ensembles from different sample batches than those used in Figure 4a (main text).

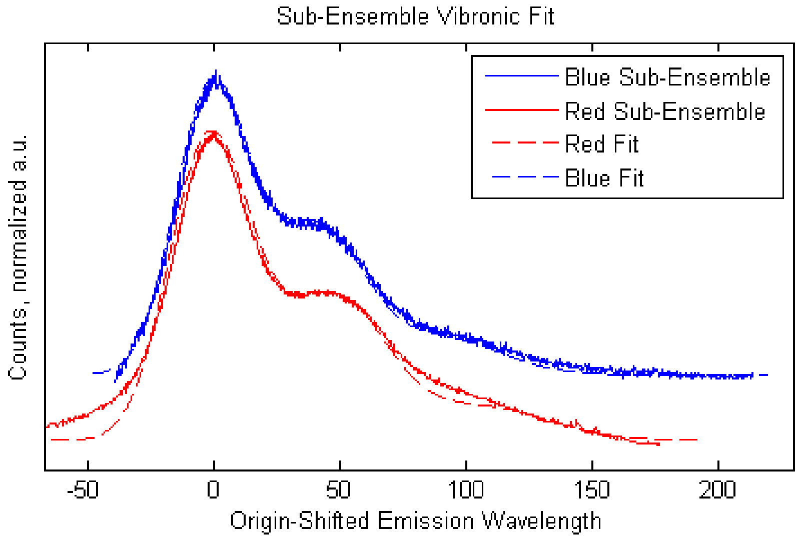

Figure S4. Vibronic fits to sub-ensembles shown in Figure S3.

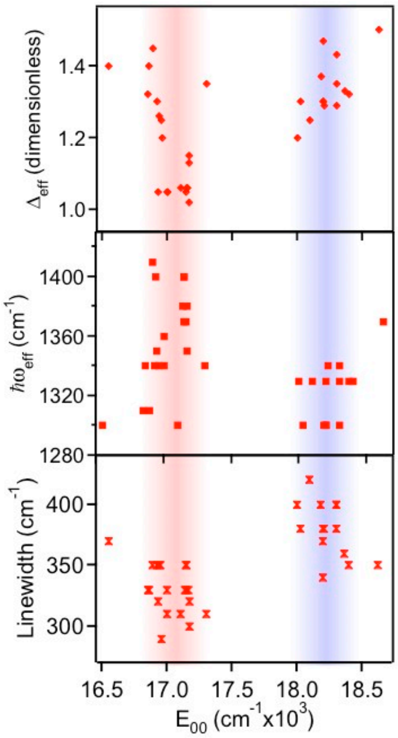

Figure S5. Comparison of $E_{0\text{-}0}$ maxima of MEH-PPV single molecule PL spectra and vibronic spectra parameters.

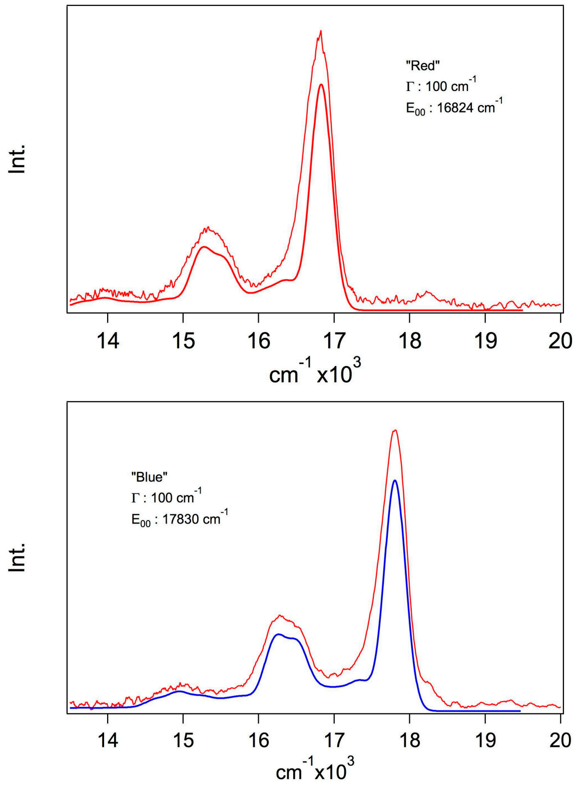

Figure S6. Low temperature (20 K) PL spectra of representative red and blue MEH-PPV emitters with line shape fit using the procedure described in the main text.

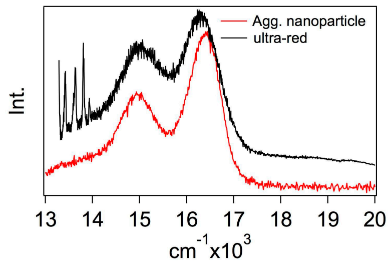

Figure S7. Comparison of PL spectra from ultra-red and aggregate nanoparticles of MEH-PPV.
